# O-GlcNAc transferase controls excitatory synapse development and AMPA receptor expression in an activity-dependent manner

**DOI:** 10.3389/fncel.2026.1799487

**Published:** 2026-03-17

**Authors:** Linkun Han, Olof Lagerlöf

**Affiliations:** 1 Department of Clinical Sciences, Umeå University, Umeå, Sweden; 2 Department of Medical Translational Biology, Umeå University, Umeå, Sweden; 3 Wallenberg Centre for Molecular Medicine, Umeå University, Umeå, Sweden

**Keywords:** AMPARs, dendritic spine, excitatory synapse, O-GlcNAc transferase, tetrodotoxin

## Abstract

Brain development and neural circuit function depend on the formation and termination of excitatory synapses. The regulation of excitatory synapse plasticity has long been associated with neuronal activity. In addition to neuronal activity, emerging data show that body metabolism affects synaptic plasticity. However, it is unclear how neuronal activity and metabolic signaling may interact to control the number and function of excitatory synapses. The nutrient sensor O-GlcNAc transferase (OGT), an enzyme that catalyzes O-GlcNAcylation of cytoplasmic and nuclear proteins depending on the metabolic state of the body, has been implicated in excitatory synapse maturation, but its activity-dependent roles and underlying mechanisms are unclear. Here, we investigated how OGT regulates excitatory synapse structure, number and AMPA-type glutamate receptors (AMPARs) in cultured hippocampal neurons under normal and activity-suppressed conditions. We show that OGT overexpression selectively enhances accumulation of the AMPARs subunit GluA1 in dendritic spines at a mature developmental stage (DIV14), but not during early development (DIV7). Chronic suppression of neuronal activity with tetrodotoxin (TTX) abolished the OGT-dependent increase in GluA1 expression, indicating that OGT-mediated regulation of AMPARs is activity-dependent. In parallel, OGT overexpression promoted coordinated growth and maturation of excitatory synapses, increasing the size and intensity of postsynaptic PSD-95 and presynaptic vGluT1 puncta, particularly at colocalized synaptic sites. These structural effects, as well as OGT-induced increases in excitatory synapse number, were eliminated by activity blockade. Together, our findings identify the nutrient sensor OGT as an activity-dependent regulator of excitatory synapse maturation and AMPARs accumulation, revealing a molecular mechanism by which neuronal activity and metabolic signaling can be integrated to shape synaptic connectivity and function.

## Introduction

Cognitive function and neural circuit development depend on the formation, maturation and termination of excitatory synapses between axons and their target neurons in the brain (Cohen-Cory, 2002; Favuzzi and Rico, 2018; Sudhof, 2021). The number and strength of excitatory synapses are not static but adapt throughout life to experience and other input that affects neuronal activity (Atwood and Karunanithi, 2002; Miehl and Gjorgjieva, 2022). Activity-dependent regulation of synaptic plasticity optimizes behavior by shaping neural circuit function according to behavioral needs (Chaudhury et al., 2016; Wright et al., 2025). In reverse, disruptions to the establishment and remodeling of excitatory synapses are implicated in neurodevelopmental, psychiatric and cognitive disorders as well as neurodegeneration (Cohen Kadosh, 2025; Tatti et al., 2017). Historically, most research on what mechanisms mediate synaptic plasticity has focused on how external changes in the environment of a person affect excitatory synapses through activity-dependent pathways. However, emerging data show that synaptic plasticity is regulated in both health and disease also by the internal environment of a person’s body. In particular, adaptive behavior depends on neuronal circuits’ constant monitoring of the body’s metabolic status to integrate environmental challenges with bodily demands (Chiel and Beer, 1997; Van Schependom et al., 2024). While it is known that synaptic plasticity depends on both neuronal activity and metabolism, the molecular mechanisms by which neuronal activity and metabolic signaling are integrated to regulate synaptic plasticity remain largely unknown.

In response to glucose and other metabolic signals, O-GlcNAc transferase (OGT) catalyzes the addition of O-linked N-acetylglucosamine (O-GlcNAc) to serine and threonine residues on cytoplasmic and nuclear proteins (Levine and Walker, 2016; Dong et al., 2025). OGT-mediated O-GlcNAcylation has emerged in neurons as an important regulator of transcription, signaling, and cytoskeletal dynamics (Chen et al., 2025; Lee et al., 2025). O-GlcNAc cycling is associated with intellectual development, neurodegenerative disorders and memory formation (Wenzel and Olivier-Van Stichelen, 2022; Lee et al., 2021). Recent studies have demonstrated that OGT regulates excitatory synapse function depending on food intake, at least in part through postprandial glucose elevation (Del Pozo et al., 2025; Andersson et al., 2021; Lagerlöf et al., 2016). However, the molecular mechanisms by which OGT regulates synapse formation and maintenance, particularly in relation to neuronal activity, remain poorly defined.

Activity-dependent synaptic plasticity critically depends on AMPA-type glutamate receptors (AMPARs), which mediate the majority of fast excitatory neurotransmission in the brain (Choquet and Hosy, 2020; de Leon-Lopez et al., 2025). The trafficking, synaptic targeting, and subunit composition of AMPARs are dynamically regulated by neuronal activity and are essential for synaptic strengthening, circuit refinement, and learning and memory (Cao et al., 2023; Henley and Wilkinson, 2016; Anggono and Huganir, 2012). Notably, previous work has shown that genetic ablation of OGT leads to reduced synaptic expression of AMPARs (Lagerlof et al., 2017), suggesting a functional link between OGT signaling and AMPAR-mediated synaptic plasticity. Whether OGT regulates AMPAR expression and synapse structure in an activity-dependent manner, however, remains unknown.

In this study, we investigated the role of OGT in regulating excitatory synapse maturation and AMPARs expression, with a particular focus on neuronal activity dependence. Using cultured hippocampal neurons, we examined how OGT overexpression influences presynaptic and postsynaptic organization under normal and activity-suppressed conditions and assessed its impact on AMPARs subunit localization across spine subtypes.

## Methods

### Animals

C57BL/6 mice were used in this study. Animals were maintained under a 12 h light–dark cycle with ad libitum access to food and water. Housing conditions were controlled at 25 °C with 50% humidity. Both male and female mice were included in all experiments. All experimental procedures were approved and conducted in accordance with the regulations of the Local Animal Ethics Committee at Umea University.

### Primary neuronal cultures

Mouse primary hippocampal neurons were prepared from embryonic day 16.5 (E16.5) embryos, as previously described (Lagerlöf et al., 2016; Ko et al., 2006). Neurons were plated on poly-L-lysine–coated coverslips at a density of 300,000 cells per well and initially maintained in NM5 medium: Neurobasal medium (Gibco, 11,570,556) supplemented with 2% B27 (v/v; Invitrogen, 15,360,284), 2 mM GlutaMAX (Gibco, 35,050–038), 5% fetal bovine serum (v/v; Cytiva, 10,309,433), and 100 U/mL penicillin/streptomycin (Gibco, 15,140–122). Two hours after plating, the medium was replaced with NM0 medium: Neurobasal medium supplemented with 2% B27 (v/v) and 5% fetal bovine serum (v/v). Cultures were maintained at 37 °C in a humidified incubator with 5% CO₂ and 95% air.

### Transfection and TTX treatment

At DIV7 or DIV14, primary hippocampal neurons plated in 12-well plates were transfected using Lipofectamine 2000 (2 μL/well; Thermo Fisher Scientific, 10,696,343) with 1.5 μg of plasmid DNA per well in Neurobasal medium supplemented with B27 (NM0; serum- and antibiotic-free). DNA and Lipofectamine were each diluted to 50 μL per well in plain Neurobasal medium and incubated separately for 5 min at room temperature. The two solutions were then combined at a 1:1 ratio and incubated for 20 min at 37 °C to allow complex formation. Transfection complexes (100 μL/well) were added to the neurons for 45 min, after which cells were rinsed with plain Neurobasal medium and returned to conditioned medium. For activity suppression experiments, tetrodotoxin (TTX, 1 μM) was added to the medium following transfection (DIV7 or DIV14), and the neurons were harvested after 24 h of TTX treatment.

### Immunocytochemistry

Cultured neurons were fixed with 4% paraformaldehyde and 4% sucrose for 10–15 min at 4 °C. Cells were then permeabilized with 0.25% Triton X-100 for 10 min at 4 °C and blocked in blocking buffer (Normal Goat Serum, Vector Laboratories, S-1000-20) for 1 h at room temperature. Neurons were incubated with primary antibodies for 2 h at room temperature, followed by washing and incubation with secondary antibodies for 1 h at room temperature. After three washes (3 × 10 min) in PBS, coverslips were mounted in Fluoromount-G and stored at 4 °C.

Primary antibodies for immunocytochemistry: anti-GFP (Aves labs, GFP-1020, 1:1000), anti- PSD95 (NeuroMab, K28/43, 1:1000), anti-vGluT1 (Millipore, AB5905, 1:1000), anti-GluA1 (NeuroMab, N355/1, 1:100), anti-Chicken secondary antibody (Azure Biosystems, AC2209, 1:2000), anti-Guinea Pig secondary antibody (Thermo Fisher Scientific, A21450, 1:2000), anti-Mouse secondary antibody (Thermo Fisher Scientific, A21422, 1:2000).

### Image acquisition and analysis

Confocal images of neuronal cultures were acquired using a Leica SP8 FALCON confocal microscope. Z-stack images covering approximately 50 μm were collected at 0.5-μm intervals. Original image files were analyzed using Imaris 10.2.1 (Bitplane). FilamentTracer was employed to reconstruct dendritic spines and assess spine density and maturation. The number and fluorescence intensity of protein marker–positive puncta were quantified, and PSD-95 and vGluT1 within GFP⁺ neurons were reconstructed in 3D and analyzed automatically using Imaris.

To ensure consistent presentation, moderate linear adjustments of contrast and brightness were applied to entire images, rather than to selected regions. All images were captured under identical acquisition settings and processed using Leica X Office software with the same parameters.

### Statistics

All statistical analyses were performed using GraphPad Prism 10 (GraphPad Software). Normality of the data was assessed when applicable. For datasets where assumptions of normality were met, student’s *t*-test was used. For multiple comparisons, Two-way ANOVA followed by Tukey’s multiple comparisons test was applied. Data are presented as individual values with mean ± SEM. Statistical significance was defined as *p <* 0.05, ****p < 0.0001; ***p < 0.001; **p < 0.01; *p < 0.05.

## Results

### The effect of OGT on GluA1 in developing and mature neurons

**Figure 1:**
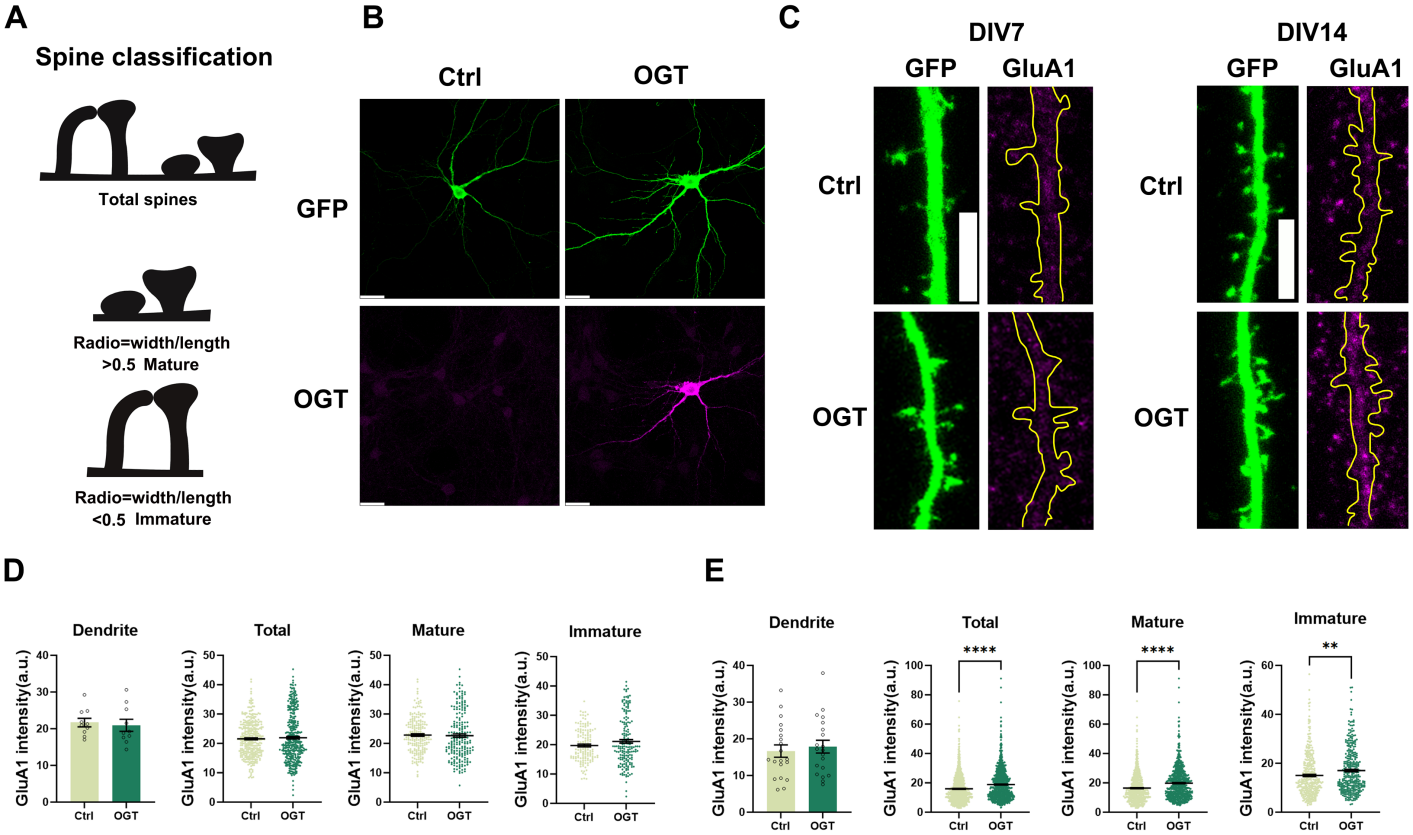
The effect of OGT on GluA1 in developing and mature neurons. **(A)** Schematic of measurements taken to quantify spine shape. **(B)** Immunohistochemistry images of GFP (green) expression in WT and OGT overexpressing hippocampal neurons transfected at DIV7. Neurons were analyzed by immunofluorescence labeling for GFP (green) and OGT (magenta). Scale bar, 20 μm (applies to all images). **(C)** Immunohistochemistry images of GFP (green) expression in WT and OGT overexpressing hippocampal neurons transfected at DIV7 (left) or DIV14 (right). Neurons were analyzed by immunofluorescence labeling for GluA1 (magenta). Scale bar, 5 μm (applies to all images). **(D)** Quantification of the expression of GluA1 in dendrite, total spines, mature spines, immature spines of individual hippocampal neurons (DIV7). Quantification was performed on spines of secondary dendrites from 10 control neurons and 10 OGT-overexpressing neurons. **(E)** Quantification of the expression of GluA1 in dendrite, total spines, mature spines, immature spines of individual hippocampal neurons (DIV14). Quantification was performed on spines of secondary dendrites from 20 control neurons and 20 OGT-overexpressing neurons. All data in this figure are presented as mean ± S. E. M. Statistical significance was determined by the unpaired Student’s *t* test (*p < 0.05, **p < 0.01, ***p < 0.001).

As the principal mediators of fast excitatory synaptic transmission in the brain, AMPA-type glutamate receptors (AMPARs) rely on tightly regulated trafficking to support excitatory signaling, synaptic plasticity, and the formation and refinement of neural circuits underlying learning and memory (Cao et al., 2023; Diaz-Alonso and Nicoll, 2021; Zheng et al., 2026). Previous studies have shown that genetic ablation of O-GlcNAc transferase (OGT) early in development leads to reduced synaptic expression of AMPARs (Lagerlof et al., 2017). However, the mechanisms by which OGT regulates AMPARs remain largely unknown. To examine whether OGT-overexpression directly influences AMPAR expression, we co-expressed GFP and OGT in cultured hippocampal immature (days-in-vitro, DIV7) or mature (DIV14) neurons and immunolabeled cells for the AMPAR subunit GluA1 (Figures 1B,C). GluA1 mediates a large part of the activity-dependent trafficking of AMPARs to and from synapses (Shepherd and Huganir, 2007; Schwarz et al., 2010). They mediate excitatory synaptic transmission on small dendritic protrusions called. Spines serve as the primary postsynaptic sites of excitatory neurotransmission and are widely regarded as the structural substrates of synaptic plasticity (Runge et al., 2020; Yasuda, 2017). Spine classification enabled quantitative analysis of GluA1 localization within total, mature, and immature dendritic spines (Figure 1A). At DIV7, Overexpression of OGT did not result in a significant difference in GluA1 expression in dendritic shaft, total spines, or in either mature or immature spine populations (Figure 1D). In contrast, at DIV14, OGT overexpression significantly increased GluA1 expression in total spines as well as in both mature and immature spines (Figure 1E), while no significant change was observed in the dendritic shaft. This pattern indicates that the elevated GluA1 signal at DIV14 primarily reflects synaptic enrichment rather than a general accumulation along the dendrite. Together, these findings suggest that OGT exerts a maturation-dependent effect on GluA1 abundance, selectively enhancing its localization to spines at later stages of neuronal development.

### OGT regulates AMPA receptor expression in an activity-dependent manner

**Figure 2:**
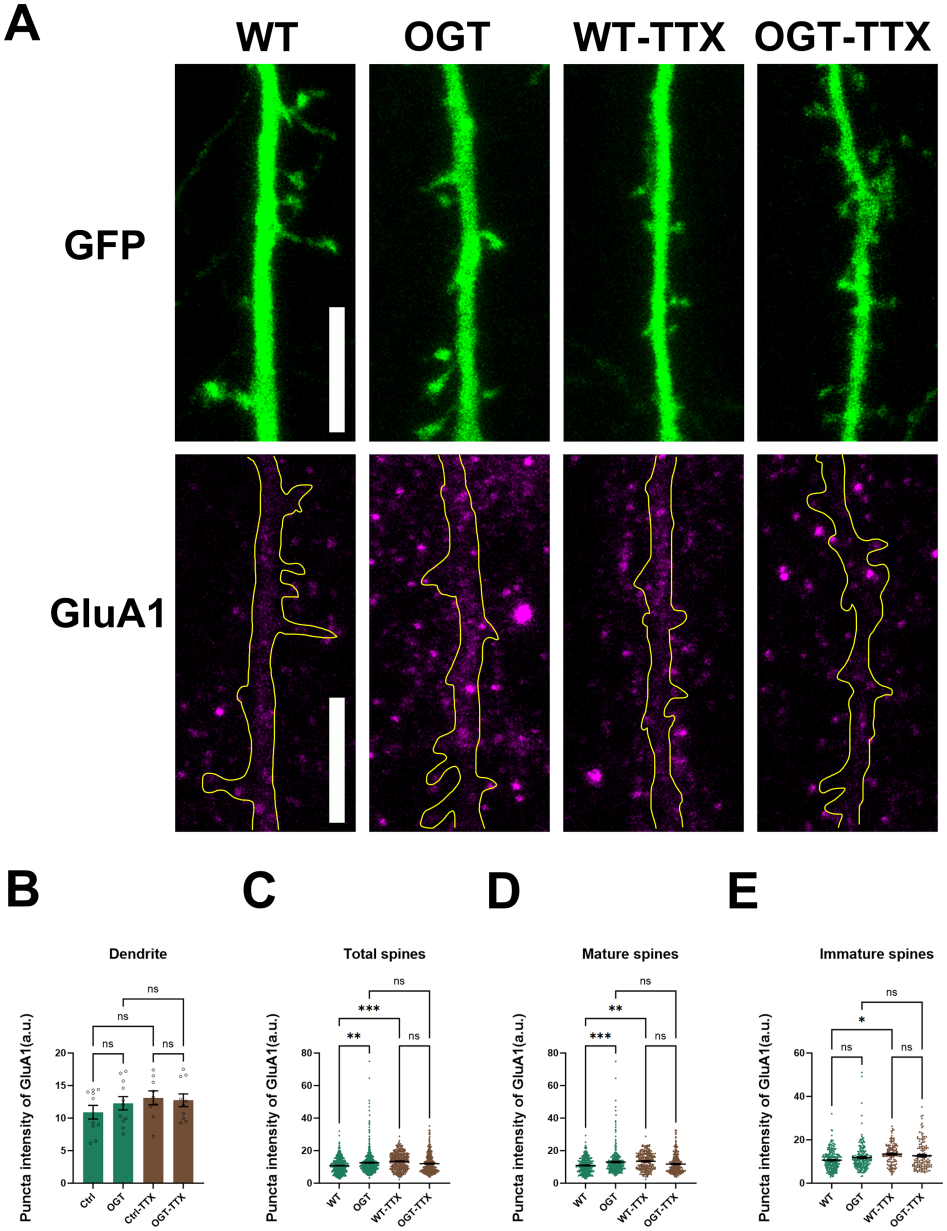
OGT regulates AMPA receptor expression in an activity-dependent manner. **(A)** Immunohistochemistry images of GFP (green) expression in WT and OGT overexpressing hippocampal neurons (with or without TTX treatment) transfected at DIV14. Neurons were analyzed by immunofluorescence labeling for GFP (green), GluA1 (magenta). Scale bar, 5 μm (applies to all images). **(B–E)** Quantification of the expression of GluA1 in dendrite **(B)**, total spines **(C)**, mature spines **(D)**, immature spines **(E)** of individual hippocampal neurons. Quantification was performed on spines of secondary dendrites from 10 WT neurons, 11 OGT-overexpressing neurons, 9 WT-TTX neurons, 10 OGT-overexpressing-TTX neurons. Data are presented as mean values ± S. E. M. Data were analyzed using a two-way ANOVA. *Post-hoc* comparisons were performed using Tukey’s multiple comparisons test (*p < 0.05, **p < 0.01, ***p < 0.001).

We also investigated whether OGT-mediated regulation of AMPARs depends on neuronal activity. To suppress neuronal activity, cultured hippocampal neurons were first transfected at DIV14. Following transfection, the neurons were treated for 24 h with tetrodotoxin (TTX), a voltage-gated sodium channel blocker. The neurons were then harvested for immunostaining experiments (Figure 2A). Under basal conditions, OGT overexpression increased GluA1 levels in both total and mature dendritic spines. In WT neurons, TTX treatment significantly increased the density of total, mature, and immature spines compared with untreated WT controls. Notably, suppression of neuronal activity completely abolished the OGT-dependent increase in GluA1 expression (Figures 2B–E). No significant difference was observed between OGT-overexpressing neurons and OGT-overexpressing neurons treated with TTX. Together, these results suggest that OGT-mediated enhancement of GluA1 expression requires neuronal activity, highlighting a critical role for OGT in coupling neuronal activity to AMPAR-dependent synaptic plasticity.

### OGT regulates the structural maturation of excitatory synapses in an activity-dependent manner

**Figure 3:**
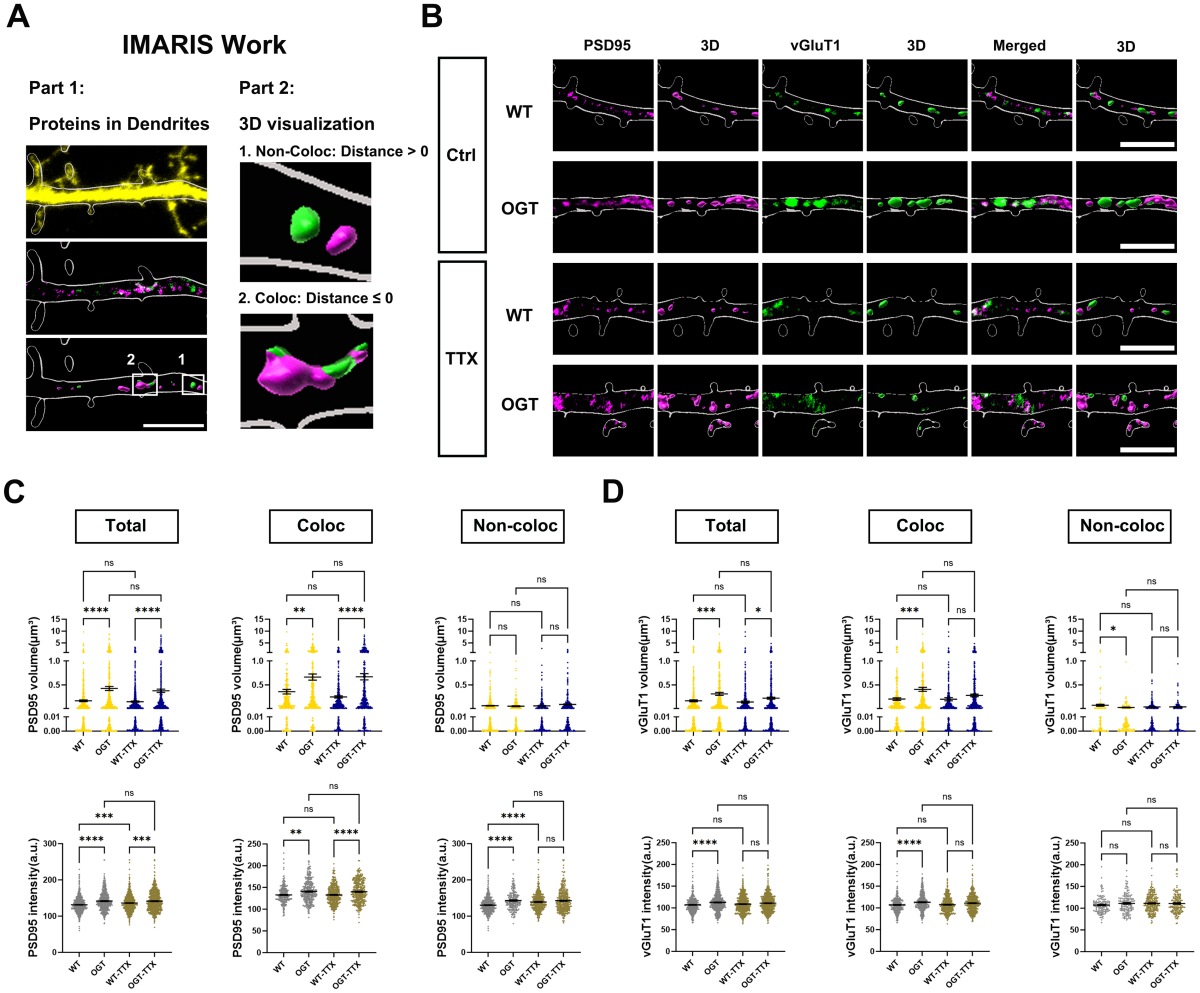
OGT regulates the structural maturation of excitatory synapses in an activity-dependent manner. **(A)** Representative 3D-rendered dendritic segment illustrating how PSD95 and vGluT1 puncta were categorized for analysis, including colocalized, non-colocalized populations. **(B)** Representative images of cultured hippocampal neurons transfected at DIV7 with expressing GFP alone (ontrol) or co expressing GFP with OGT (OGT overexpression) with or without TTX treatment. Neurons were analyzed by triple immunofluorescence labeling for GFP (outline), PSD95 (magenta), and vGluT1 (green). Scale bar, 5 μm (applies to all images). **(C,D)** Quantification graphs of the effects of OGT overexpression in neurons on size **(C)** and intensity **(D)** of PSD95 or vGluT1 puncta. All data in this figure are presented as mean ± S. E. M. Data were analyzed using a two-way ANOVA. *Post-hoc* comparisons were performed using Tukey’s multiple comparisons test (*p < 0.05, **p < 0.01, ***p < 0.001).

Our observation that OGT regulates activity-dependent abundance of AMPARs in dendritic spines, which are widely regarded as the structural substrates of excitatory synaptic plasticity, suggested to us that OGT may regulate synapse structure and number depending on neuronal activity (Hering and Sheng, 2001; Minegishi et al., 2023). The precise formation of synaptic connections between developing axons and their appropriate targets is essential for proper nervous system function (Cohen-Cory, 2002; Chao et al., 2009). Previous studies have demonstrated that O-GlcNAc transferase (OGT) plays a role in regulating excitatory synapse maturation (Lagerlof et al., 2017). However, the underlying mechanisms remain poorly understood. To investigate this mechanism, we co-expressed GFP and OGT in cultured hippocampal neurons as before (Figure 3B). Neuronal activity was chronically suppressed by treating cultures with tetrodotoxin (TTX) for 24 h. Neurons were then immunolabeled with antibodies against the excitatory presynaptic marker vGluT1 and the postsynaptic marker PSD95 (Figure 3B). Using Imaris software, we quantified the number, volume, and fluorescence intensity of vGluT1 and PSD95 puncta within dendrites. In addition, synaptic colocalization was assessed based on the spatial proximity of presynaptic and postsynaptic puncta (Figure 3A). Puncta were categorized as Total (all puncta within the dendrite), Coloc (puncta colocalized with a corresponding punctum of the other marker) and Non-coloc (puncta without colocalization).

Overexpression of OGT resulted in a significant increase in the size and intensity of PSD95 (Total or Coloc), but not in the size of Non-coloc PSD95 puncta (Figure 3C). No significant differences in PSD-95 puncta size were observed between TTX-treated neurons and untreated neurons (Figure 3C). However, in WT neurons, TTX treatment significantly increased PSD95 puncta intensity in the Total and Non-coloc categories, with no change observed in the Coloc population (Figure 3C). No significant differences were detected between OGT-overexpressing neurons and OGT-overexpressing neurons treated with TTX. Notably, the OGT-induced increase in Non-coloc PSD95 intensity was eliminated following TTX treatment (Figure 3C). We then examined presynaptic vGluT1 labeling. No significant differences in vGluT1 puncta volume or size were observed between WT neurons and WT neurons treated with TTX, nor between OGT-overexpressing neurons and OGT-overexpressing neurons treated with TTX. Analysis of vGluT1 labeling revealed an increase in Total vGluT1 puncta size under both control and TTX conditions (Figure 3D), whereas increases in Coloc vGluT1 puncta size and in Total and Coloc vGluT1 puncta intensity were eliminated by activity blockade (Figure 3D). In contrast, OGT decreased the vGluT1 volume in non-colocalized puncta. This decrease was abolished upon blocking neuronal activity (Figure 3D). Together, these results demonstrate that elevated OGT expression promotes structural growth and maturation of excitatory synapses, and that many of these effects require ongoing neuronal activity.

### OGT enhances excitatory synapse number via neuronal activity

**Figure 4:**
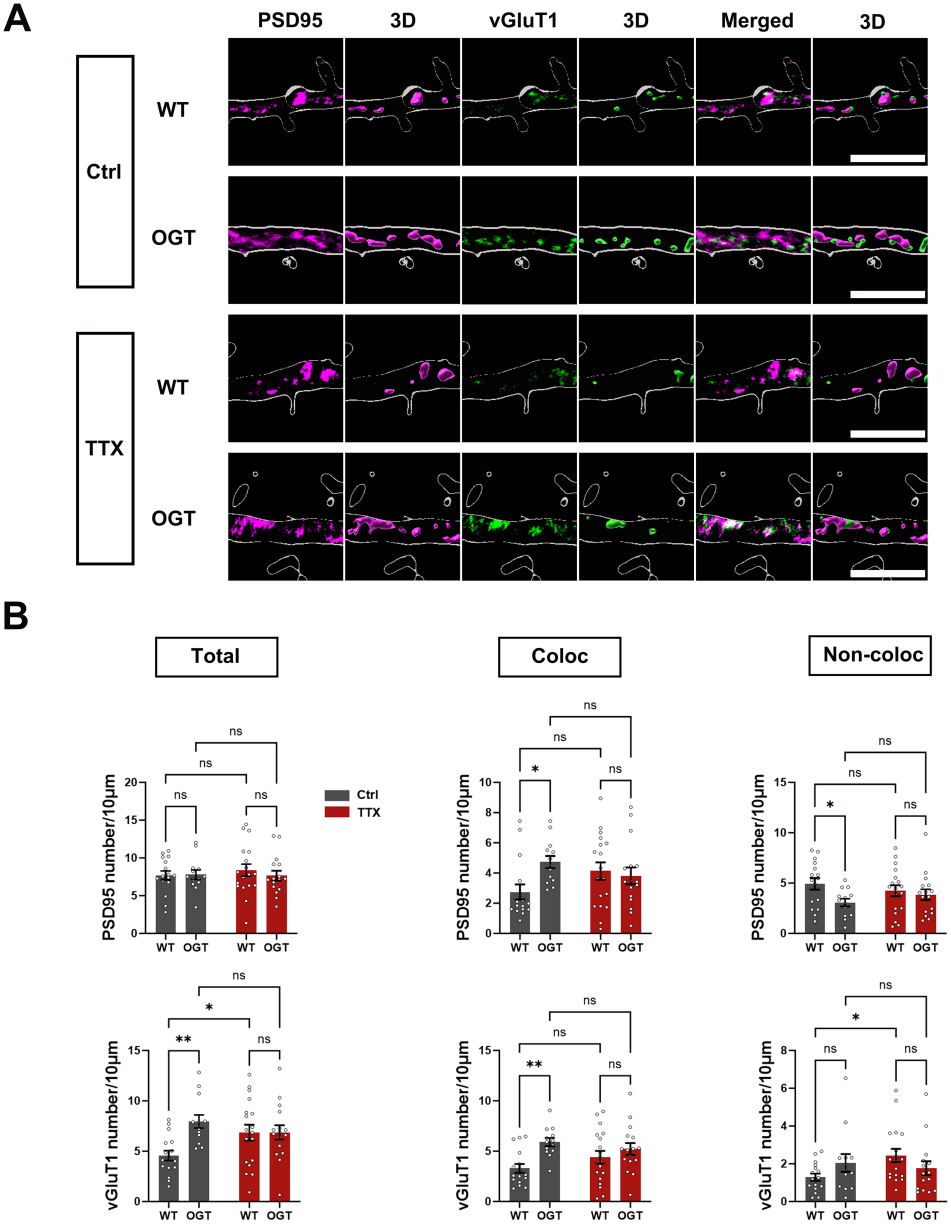
OGT enhances excitatory synapse number via neuronal activity. **(A)** Representative images of cultured hippocampal neurons transfected at DIV7 with expressing GFP alone (control) or co expressing GFP with OGT (OGT overexpression) with or without TTX treatment. Neurons were analyzed by immunofluorescence labeling for PSD95 (magenta), and vGluT1 (green). Scale bar, 5 μm (applies to all images). **(B)** Quantification graphs of the effects of OGT overexpression in neurons on density of PSD95 or vGluT1 puncta. Quantification was performed on puncta in secondary dendrites from 18 WT neurons, 16 OGT-overexpressing neurons, 18 WT-TTX neurons, 16 OGT-overexpressing-TTX neurons. All data in this figure are presented as mean ± S. E. M. Statistical significance was determined by the two-way ANOVA test (*p < 0.05, **p < 0.01, ***p < 0.001).

To determine whether OGT also influences synapse number, we quantified the number of PSD95 and vGluT1 puncta per 10 microns along dendrites (their density) (Figure 4A). In untreated cultures, OGT overexpression significantly increased the number of colocalized PSD95 puncta and decreased the number of non-colocalized PSD95 puncta relative to controls. Both effects were abolished by TTX treatment (Figure 2B). No significant differences in the number of PSD95 puncta were observed between WT neurons and WT neurons treated with TTX, nor between OGT-overexpressing neurons and OGT-overexpressing neurons treated with TTX. Similarly, OGT overexpression significantly increased total and colocalized vGluT1 puncta number in control conditions, an effect that also was eliminated by activity suppression (Figure 2B). In WT neurons, TTX significantly altered the number of total and non-colocalized vGluT1 puncta. However, no differences were detected between OGT-overexpressing neurons and OGT-overexpressing neurons treated with TTX. These results demonstrate that OGT enhances excitatory synapse number in a neuronal activity–dependent manner.

**Figure 5:**
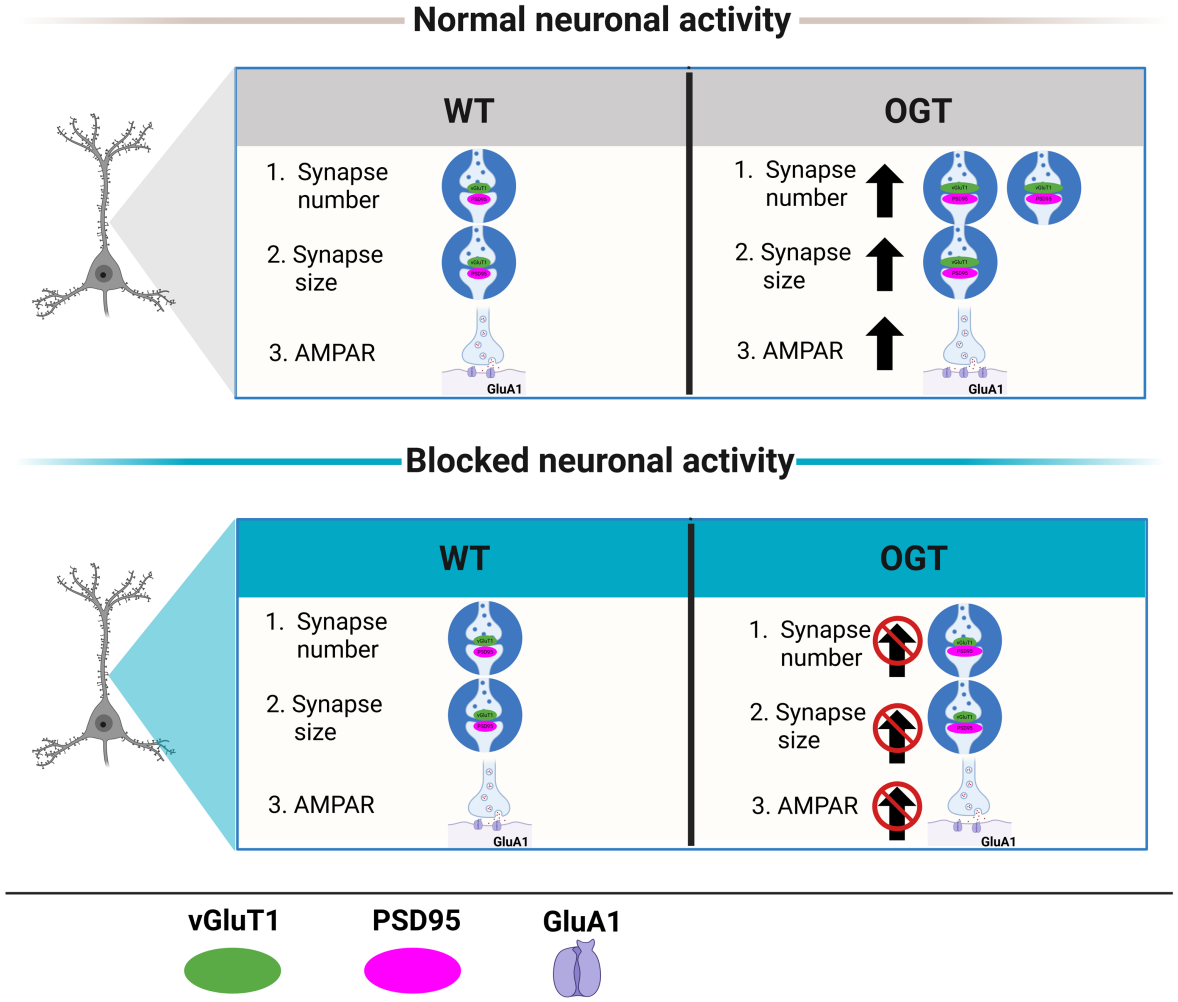
A model of OGT acts as an activity-dependent regulator of excitatory synapse maturation. Schematic illustrating that O-GlcNAc transferase (OGT) promotes excitatory synapse maturation and AMPA receptor accumulation in an activity-dependent manner. Chronic neuronal activity blockade with tetrodotoxin (TTX) abolishes these OGT-mediated effects, indicating that neuronal activity is required for OGT-dependent synaptic development.

Together, our findings support a model in which OGT is an activity-dependent regulator of excitatory synapse maturation (Figure 5). Under normal neuronal activity, OGT enhances accumulation of the AMPARs subunit GluA1 in dendritic spines. Elevated OGT promotes coordinated growth of presynaptic and postsynaptic compartments, increasing vGluT1 and PSD95 puncta size, intensity, colocalization, and excitatory synapse number. In contrast, chronic neuronal activity blockade with TTX abolishes these OGT-mediated effects on GluA1 expression, synaptic growth and synapse number.

## Discussion

In this study, we identify O-GlcNAc transferase (OGT) as a critical, activity-dependent regulator of excitatory synapse maturation and AMPA receptor accumulation in hippocampal neurons. By combining analyses of GluA1 localization, synaptic marker organization, and synapse number under conditions of altered OGT expression and neuronal activity, our findings provide mechanistic insight into how OGT links metabolic and activity-dependent signaling to structural and functional synaptic plasticity.

A key finding is that OGT overexpression selectively increases GluA1 accumulation within dendritic spines at a mature developmental stage (DIV14), without affecting levels in the dendritic shaft. This effect is not observed at an earlier stage (DIV7). The selective enhancement at DIV14 indicates that the increased GluA1 signal reflects synaptic enrichment rather than a nonspecific accumulation along the dendrite. This developmental specificity further suggests that OGT does not constitutively upregulate AMPAR expression but instead regulates receptor trafficking or stabilization in a maturation-dependent manner. Early in development, when synapses are highly dynamic and immature, AMPAR recruitment may be governed predominantly by intrinsic developmental programs or baseline activity levels. As neurons mature and synaptic networks stabilize, OGT may become increasingly important for fine-tuning AMPARs content at synapses, thereby contributing to experience-dependent refinement of excitatory circuits. This temporal distinction is consistent with previous reports based on genetic deletion that OGT is required for normal synaptic AMPARs expression (Lagerlof et al., 2017). These data together with a recent observation showing that decreasing O-GlcNAc by overexpressing OGA, the enzyme that removes O-GlcNAc from proteins, lead to the opposite effect on GluA1 in spines suggest strongly that OGT’s effect on GluA1 is bidirectionally mediated through changing O-GlcNAc levels (Han et al., 2026). Hence, our results indicate that elevated OGT by increasing O-GlcNAcylation can actively promote GluA1 enrichment at spines in a stage-dependent manner.

Our results further reveal that OGT-dependent regulation of GluA1 is tightly coupled to neuronal activity. Chronic blockade of action potential firing with TTX abolished the enhancement of GluA1 expression induced by OGT overexpression. These findings indicate that OGT does not act independently of synaptic activity, rather, it functions as a permissive or amplifying factor that requires ongoing neuronal firing to exert its effects on AMPAR localization. Nevertheless, chronic TTX treatment is known to induce homeostatic synaptic scaling, which increases synaptic AMPAR abundance. Under these conditions, it is theoretically possible that AMPAR levels at synapses may approach a functional ceiling, potentially masking additional effects of OGT overexpression on GluA1 accumulation or synaptic protein colocalization. However, in our experiments, the TTX-treated group exhibited a clearly distinct trend toward decreasing GluA1 levels in both mature and immature spines upon overexpressing OGT. This strengthens the conclusion that OGT’s effect on AMPA receptors is activity dependent. It is also the case that previous reports using electrophysiology indicate that OGT regulates LTP, a finding that further supports our results on the molecular regulation of excitatory synapses by OGT (Taylor et al., 2014). Future experiments using, e.g., acute TTX exposure or chemically induced LTP, which minimizes homeostatic compensation, and OGT loss-of-function approaches would help clarify the mechanism by which OGT modulates AMPAR trafficking.

The regulation of AMPAR levels in spines is linked to activity-dependent regulation of synapse size and number (Cao et al., 2023; Antunes and Simoes-de-Souza, 2018). Indeed, overexpression of OGT selectively increased the size and intensity of PSD-95 puncta at colocalized synaptic sites, while non-colocalized PSD95 puncta were largely unaffected. This suggests that OGT preferentially stabilizes or strengthens existing synaptic contacts rather than indiscriminately increasing postsynaptic protein accumulation. Similarly, the observed increases in vGluT1 puncta size and intensity further support a coordinated enhancement of presynaptic and postsynaptic components during synaptic maturation. The fact that many of these effects were abolished by chronic TTX treatment indicates that neuronal activity is required for OGT-mediated synaptic remodeling. Consistent with these structural changes, OGT overexpression also increased excitatory synapse number, as measured by the density of colocalized PSD95 and vGluT1 puncta. The elimination of this effect by TTX underscores the need of neuronal activity for OGT-driven synaptogenesis or synapse stabilization. Many observations in conditions associated with impaired synapses such as autism and schizophrenia indicate that neuronal circuit dysfunction is not simply a function of either too many or too few synapses but rather how synapses are shaped and numbered based on neuronal activity (Lepeta et al., 2016; Bonsi et al., 2022; Zoghbi and Bear, 2012).

Functionally, our findings place OGT at the intersection of metabolic sensing, neuronal activity, and synaptic plasticity. Given that O-GlcNAcylation is sensitive to cellular nutrient status, OGT may provide a molecular mechanism by which metabolic state influences the capacity for synaptic growth and plasticity. This has potential implications for neurodevelopmental and neurodegenerative disorders in which both metabolic dysregulation and synaptic dysfunction are prominent. OGT is linked in humans to both impaired neurocognitive development and neurodegenerative disorders. In these conditions, altered OGT activity could contribute to impaired synapse maturation, reduced AMPAR expression, and defective circuit refinement, ultimately affecting learning and memory.

While the present study focuses on excitatory glutamatergic synapses and AMPAR regulation, future investigations should expand this framework to additional components of synaptic transmission. In particular, it will be important to determine whether OGT similarly modulates N-methyl-D-aspartate (NMDA) receptor composition, trafficking, or function under comparable activity paradigms, given the central role of NMDA receptors in synaptic plasticity. Examining NMDA receptor-dependent signaling and plasticity would provide a more comprehensive understanding of how OGT influences synaptic maturation and circuit refinement. In addition, future studies should address whether OGT regulates inhibitory synapses under similar conditions. Because the coordinated development of excitatory and inhibitory synapses is essential for maintaining proper circuit function and stability, determining whether OGT affects GABAergic receptor trafficking, inhibitory synapse formation, or inhibitory synaptic strength will be critical. Such investigations will clarify whether OGT serves as a broader regulator of synaptic balance and network maturation or instead exerts selective effects on specific components of excitatory circuitry.

## Conclusion

In conclusion, our results demonstrate that OGT promotes the structural maturation of excitatory synapses, increases synapse number, and enhances AMPAR accumulation in an activity-dependent manner. These findings position OGT as a critical regulator of synaptic development and plasticity, with potential implications for understanding how metabolic and signaling states influence neuronal circuit formation and function. Further elucidation of OGT’s synaptic substrates and regulatory pathways may provide new insight into the molecular mechanisms underlying learning, memory, and neurodevelopmental disorders associated with dysregulated synaptic plasticity.

## Data Availability

The raw data supporting the conclusions of this article will be made available by the authors, without undue reservation.
